# An Examination of Knowledge, Attitudes, and Intentions Related to Human Papillomavirus (HPV) Vaccination Among Undergraduate Students in Saint Lucia

**DOI:** 10.7759/cureus.81426

**Published:** 2025-03-29

**Authors:** Esther S Daniel, Tisha Nelson, Gloria Ramdeen-Mootoo, Abdulqadir J Nashwan, Parbatee Siewdass, Virginia Mary

**Affiliations:** 1 Epidemiology, Faculty of Medical Sciences, The University of the West Indies (UWI) School of Nursing, St. Augustine, TTO; 2 Nursing, Sir Arthur Lewis Community College, Castries, LCA; 3 Clinical Psychology, Faculty of Medical Sciences, The University of the West Indies (UWI) School of Nursing, St. Augustine, TTO; 4 Nursing and Midwifery Research, Hazm Mebaireek General Hospital (HMGH), Doha, QAT; 5 Oncology, Faculty of Medical Sciences, The University of the West Indies (UWI) School of Nursing, St. Augustine, TTO; 6 Psychiatry, Faculty of Medical Sciences, The University of the West Indies (UWI) School of Nursing, St. Augustine, TTO

**Keywords:** cervical cancer, hpv vaccination, human papilloma virus, pap smear, sexually transmitted infection (sti)

## Abstract

Objective: In the Caribbean, cancer ranks as the second leading cause of mortality. Without interventions, the incidence is predicted to increase by 66% within the next decade, with over 56,000 women being diagnosed with cervical cancer annually and more than 28,000 succumbing to the disease. However, lifesaving prevention and treatment measures are available. This research sought to explore potential differences in knowledge, attitudes, and willingness to receive human papillomavirus (HPV) vaccinations among male and female undergraduate students at a chosen Community College in St. Lucia.

Methods: This investigation utilized a descriptive, quantitative, and cross-sectional approach. Participants (n = 100) were chosen through random sampling and completed 59-item self-administered questionnaires addressing the targeted variables. Multivariable statistical techniques were employed to determine independent predictors of HPV vaccine acceptance among participants.

Results: Despite recognizing the associated risks, the findings highlighted a substantial deficit in public health promotion efforts. First, although HPV is widespread in St. Lucia and causes cancer in both genders, there is insufficient awareness-raising about HPV. Second, the vaccine is not accessible in St. Lucia. Third, the high expenses associated with the vaccine ($500 US) and HPV test ($275 EC) hinder obtaining them. Finally, HPV vaccine uptake was minimal among participants; only seven out of 100 had been vaccinated and received it in the US. Despite low vaccine uptake, 53% acknowledged the urgent need for vaccination.

Conclusions: Integrating HPV vaccination within healthcare and HPV awareness initiatives can yield positive outcomes in mitigating cancer's long-term impact on St. Lucia's male and female populations. Educational programs could prove advantageous in enhancing knowledge about HPV and vaccinations.

## Introduction

In St. Lucia, no research has been carried out on human papillomavirus (HPV), with a cancer registry only being established in the Ministry of Health's Epidemiological Department in early 2016. Various sectors, including the public and private sectors, local laboratories, and private hospitals, have recognized HPV's prevalence. The HPV test was introduced by a private laboratory in 2015, and samples were sent to Jamaica for analysis. Consequently, significant delays have arisen in receiving test results, sometimes taking up to six months [1].

The St. Lucia Public Health Act 1975 mandates that the Minister of the Ministry of Health be directly responsible for preventing, treating, limiting, and suppressing illnesses, as well as conducting relevant research and inquiries [1]. However, it is assumed that the Ministry of Health of St. Lucia has not made adequate decisions regarding the provision of the HPV vaccine and informing parents about HPV and the vaccine. The unavailability of the HPV vaccine has socioeconomic implications for the population and the country as a whole.

When an individual develops cancer due to HPV infection, the high cost of chemotherapy and treatment regimens can lead to financial strain on both the individual and the nation, as they may be unable to work or experience reduced productivity due to illness. As the number of infected individuals rises, so does the financial burden on the country's economy. By studying knowledge, attitudes, and intentions regarding HPV, health educators can gain insights into perceived barriers to immunization in this community by examining college students' vaccination rates. The researcher's extensive nursing experience has highlighted the urgent need for this investigation, given the high prevalence of HPV-related cancers in St. Lucia. This study explored potential differences in knowledge, attitudes, and willingness to receive HPV vaccinations among male and female undergraduate students at a selected Community College in St. Lucia.

Young women around the world still face a substantial burden from cervical cancer, especially in low- and middle-income countries. Limited data exist on the number of young women in Latin America and the Caribbean (LAC) who succumb to cervical cancer. The World Health Organization's mortality database provided information on cervical cancer fatalities. Age-standardized death rates per 100,000 women-years were determined for women aged 20-44 in 16 countries (and territories) across LAC from 1997 to 2017, using the world standard population [2].

Between 2014 and 2017, Puerto Rico exhibited the lowest cervical cancer mortality rates, while Paraguay and Venezuela recorded the highest rates. Cervical cancer mortality generally decreased in most LAC countries throughout the period. Average annual percent change (AAPC) calculations showed significant reductions in Chile (-2.4%), Colombia (-2.0%), Cuba (-3.6%), El Salvador (-3.1%), Mexico (-3.9%), Nicaragua (-1.7%), Panama (-1.7%), and Peru (-2.2%). Conversely, Brazil showed an AAPC of +0.8%. On the other hand, Paraguay (+3.7%) demonstrated notable positive trends. By 2030, death rates are not anticipated to decline further in several LAC countries, such as Argentina, Paraguay, and Venezuela [2].

Only a handful of studies have been conducted in the Caribbean, specifically in Grenada, Puerto Rico, Latin American territories, and The Bahamas. Numerous investigations on HPV and cervical cancer, oral cancer, Epstein-Barr Virus, HPV knowledge, and HPV vaccination have been conducted in other countries, such as Sweden, China, Denmark, Turkey, Nigeria, Haiti, Thailand, Germany, and the US [3].

Despite the high cervical cancer risk in the Caribbean, there has been minimal research on the virus and HPV vaccination in the region. The "Getting to Zero" HPV study, conducted in The Bahamas in 2014/2015 with a sample size of 1,553, offered cross-sectional data. Merely 10.7% (146/1,364) of the participants were aware of HPV. Among those who reported being sexually active (n = 685), only 10.7% had heard of HPV [3]. To achieve effective HPV vaccination uptake, it emphasizes the importance of developing a comprehensive implementation plan that evaluates knowledge and attitudes [3].

In LAC, there are approximately 1.5 million new cancer cases per year and 700,000 deaths, with incidence and mortality rates of 186.5 and 86.6 per 100,000, respectively. In 2020, the most prevalent cancers were prostate (15%), breast (14%), colorectal (9%), lung (7%), and stomach (5%). Lung cancer remained the leading cause of cancer-related death (12%), although mortality rates varied significantly between countries. While cancer rates linked to westernization generally increased, mortality trends for cancers related to infectious diseases tended to decline in many countries. Assuming that rates remain unchanged, by 2040, LAC would witness 2.4 million new cancer cases annually, reflecting a 67% increase [4].

Worldwide, the HPV infection is among the most prevalent sexually transmitted infections. Annually, 20-80 women per 100,000 are diagnosed with cervical cancer, resulting in the LAC having some of the highest incidence and mortality rates globally. Only 10% of sexually active adolescents and 46% of adults were aware of the HPV [1].

In 44 countries and territories, 27 (61.3%) have the HPV vaccine in their national immunization program. GNV programs have been implemented by eight countries, mainly via a mixed strategy (school-based and health centers). The recommendation to incorporate HPV immunization for men habitually occurred more than a few years after the early introduction of female vaccination: Argentina (2011/2017), Barbados (2014/2016), Bermuda (2011/2016), Brazil (2014/2017), Panama (2008/2016), Puerto Rico (2007/ 2011) and Trinidad and Tobago (2013/2014) [4].

After the successful launch of the HPV immunization program, concerns about vaccine safety impacted public opinion and media coverage. Heightened media attention may have contributed to the decline in vaccination uptake, as suggested by the apparent change in the relationship between vaccination uptake and media coverage before and after July 2013 [4]. A study carried out in Spain suggested that vaccinating all Spanish teenagers would be valuable if the potential protection against other cancers or a reduction in the vaccine's cost per dose were considered [5]. Of the 313 male university students who had heard of HPV, 59.42% were knowledgeable about HPV and related diseases; among the 300 male university students who were aware of HPV vaccines, 75.33% were informed [6].

In Turkey, 552 parents consisted of 438 women and 114 fathers. The majority of parents (69%) confessed to being unaware of HPV vaccinations. Among 532 parents with daughters, 76.7% favored immunization, and 74.6% of the 508 parents with sons also supported it. After being informed about the cost of the vaccines and the number of required doses, 11.2% of parents (n = 62) stated they would not vaccinate their children even if the government provided the vaccine for free [7].

Most medical students did not perceive themselves as susceptible to HPV, with only 8% of female students and none of the male students having received the HPV vaccine. The notion that HPV vaccination might promote sexual activity among young people was more common among male students than female students (35% vs. 15%; p < 0.001) [8]. In a study involving 144 students, 71% were female. A total of 37.5% of participants reported learning about HPV through social media. Among them, 82% of women and 25% of men possessed knowledge about HPV [9].

In Lebanon, the average age for receiving the first dose of the vaccine was 17.5 years. Moreover, 48% of respondents indicated they would not obtain the HPV vaccine in the upcoming year. A student enrolled in a public university had a 77% probability of not getting immunized. Additionally, female students with a father having an educational background beyond a university degree showed an 88% vaccination rate. For every point increase in knowledge of the vaccine, the likelihood of getting an HPV vaccination rose by 37% [10].

In India, a survey of 988 students found that 95% of them had some knowledge about genital warts, HPV, or cervical cancer (77.5). Before the study, only 59.7% of respondents were aware of the HPV vaccine; 65.2% intended to receive it, and 68.3% would likely recommend it to others. Participants under 22 years old were less inclined to agree to the vaccination [11].

When asked about their comprehension of the HPV vaccine, approximately 267 (62.2%) participants stated that there was no HPV vaccination, and 239 (61.9%) respondents believed that the vaccine did not lower the likelihood of cervical cancer. Moreover, the likelihood of receiving an HPV vaccine on a physician's recommendation was considerably higher than on a friend's recommendation (relative importance index, RII = 0.745 vs. 0.675), according to the participants [12].

This article was initially posted on the Research Square preprint server on April 25, 2023.

## Materials and methods

Conceptual framework

**Figure 1:**
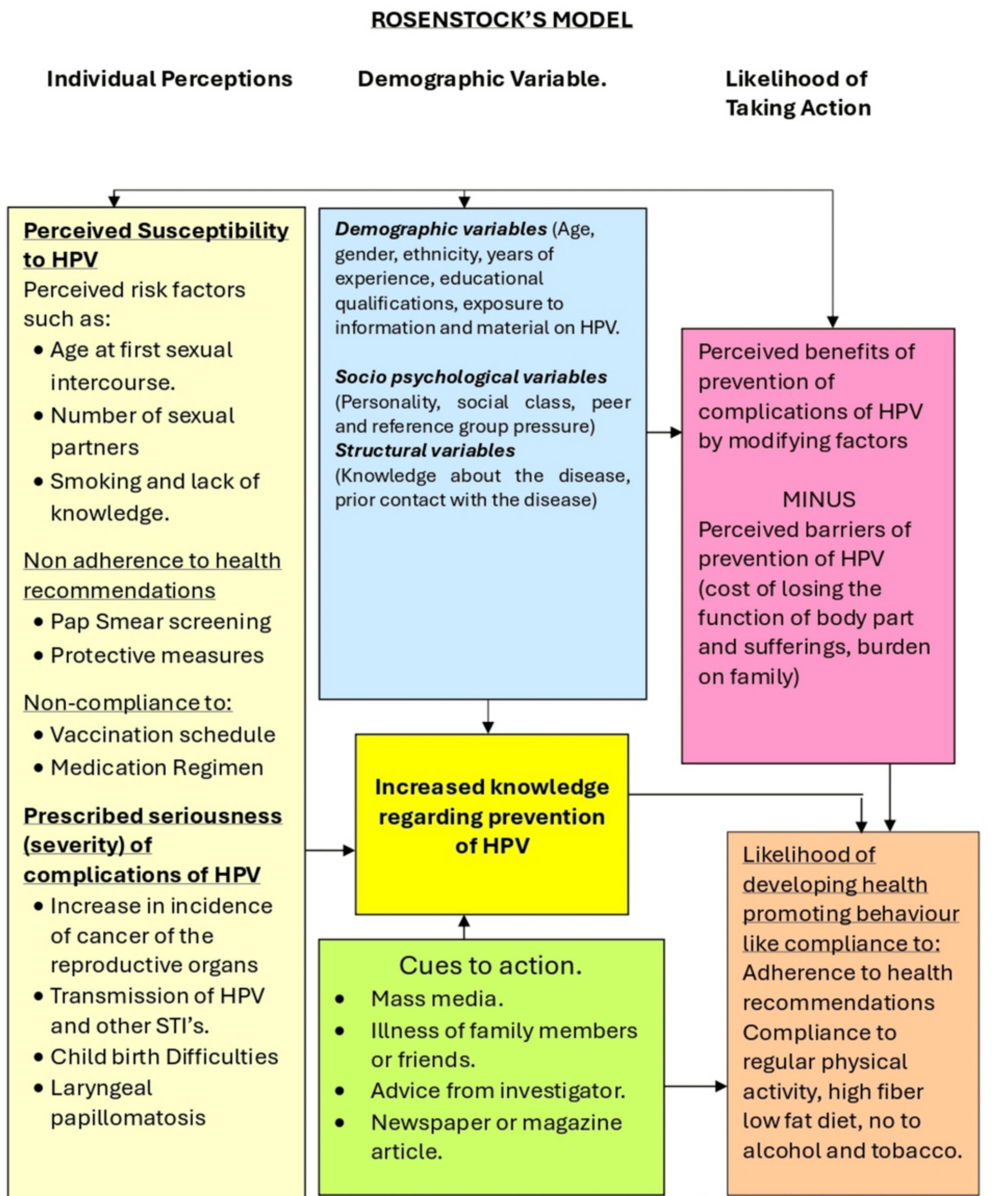
Conceptual framework HPV: human papillomavirus; STI: sexually transmitted infection Image credits: This is an original image created by the author Esther S. Daniel

The Health Belief Model (HBM) was utilized as a framework for this study. The HBM is based on the premise that health-seeking behavior is highly influenced by an individual's perception of a threat posing a health problem and the value attributed to actions that reduce the threat (Figure 1). Applying theories like the HBM necessitates filling in knowledge and awareness gaps among individuals on risk, vulnerability, and efficacy to deal with health issues [1].

The HBM was first developed in the 1950s to predict whether people will participate in campaigns to identify and stop sickness [13,14]. The HBM consists of five elements designed to influence a person's propensity to adopt a particular health behavior to avoid a negative health consequence. They include perceived susceptibility (perceptions of the likelihood of experiencing the outcome), perceived severity (perceptions of the gravity of the consequences associated with the outcome), perceived benefits (possible advantages of engaging in the health behavior, including the behaviors’ perceived efficacy in preventing the undesirable outcome), and perceived barriers (perceived obstacles to engaging in the health behavior). The fifth and least explored component of the HBM, known as "cues to action," refers to internal or external stimuli that prompt individuals to engage in health-promoting behaviors. These components may involve experiencing symptoms or hearing persuasive justifications from members of the public, close friends, or medical experts [13,14].

The majority of the studies (77%, k = 54), the majority of which focused on women, used readily available samples (80%, k = 56), and only 47% (47%, k = 33) did not employ any theories. In theory-driven studies, framing (22%, k = 19), the HBM (13%, k = 12), and narrative (7%, k = 6) were the three most often used theories.

Promotional strategies significantly outperformed controls in controlled studies (r+ =0.25, p = 0.001). Strategies guided by the information, motivation, and behavioral skills model were more effective than those guided by the framing theory (r+ = 0.75, p = 0.001), the HBM (r+ = 0.01, p = 0.001), and other theories (r+ = 0.11, p = 0.001) [15].

The data analysis was based on the research question: What is the knowledge, attitude, and intention to obtain HPV vaccination among the male and female undergraduates at the selected Community College in St. Lucia? Are there differences in knowledge, attitude, and intention to obtain the HPV vaccine that may impact one's likelihood of seeking medical aid? How informed are the subjects on HPV vaccination and its effectiveness in preventing cancer (cervical, oral) at the Sir Arthur Lewis Community College in St. Lucia? This study aimed to investigate whether there were any variations in knowledge, attitude, and desire to vaccinate against HPV among the male and female undergraduates of a selected Community College in St. Lucia (Figure 1).

Methods

Study Setting

The study was conducted at Sir Arthur Lewis Community College, located at Morne Fortune, Castries, on the beautiful Island of St. Lucia.

Study Design

This is a descriptive, quantitative, and cross-sectional study.

Participants and Sample Size

The population consisted of undergraduate students at a community college in Saint Lucia. The sample (n = 140) was selected using stratified random sampling.

Sampling

A random stratified sample of college students between the ages of 16 and 29 years enrolled at the Sir Arthur Lewis Community College in St. Lucia, in the second and third semesters of 2016. The study began on September 18, 2015, and concluded on August 20, 2016. The samples were selected in the following manner.

Step 1: Initially, the entire lists of students studying in the selected college were obtained from the Principal.

Step 2: A second list was prepared from the college departments, namely the Division of Arts, Science, and General Studies, the Department of Health Sciences, the Department of Agriculture, the Department of Teacher Education, and the Department of Technical and Management Studies. This made a total of five departments.

Step 3: A third list consisting of students from this department was obtained from the Principal/Coordinator. The Division of Arts, Science, and General Studies; Department of Teacher Education; and Division of Technical and Management Studies were selected randomly.

Step 4: From this list, 10% of the samples were drawn using random tables to select samples.

Step 5: A total sample size of 140 students was selected for this study. Only 100 students volunteered to participate in the study.

The inclusion criteria for sample selection included male or female respondents, between the ages of 18 and 26, enrolled in undergraduate or diploma programs, belonged to the Health Sciences Department, represented various ethnicities and races as reported by the respondents, had a range of relationship statuses (single, married, in a relationship, etc.), and were willing to participate voluntarily. Exclusion criteria were part-time respondents and online students who did not consent to the study.

Data Collection

A self-administered, structured tool was developed to effectively assess knowledge, attitudes, and intention to receive the HPV vaccine. Regarding its development and validation, the questionnaire was designed based on an extensive review of relevant literature and tailored to address the specific objectives of the study.

To ensure its validity and reliability, the questionnaire underwent pilot testing with a small group of participants who were representatives of the study sample. Feedback from this pilot test was used to refine the questions for clarity and consistency. Additionally, the questionnaire was reviewed by experts in the field to assess its content validity, ensuring that it accurately measured the constructs intended.

A self-administered questionnaire was utilized to collect demographic data (10 items), a knowledge questionnaire (24 items), an attitudes scale (10 items), and a Likert scale measuring intentions to obtain HPV (15 items). Variables of study included knowledge, attitude, and intention to obtain HPV. Data were verified and coded by the researcher (see the questionnaire in the Appendix).

Section A consisted of participants’ demographic characteristics: age, gender: male/female; current year in college: first year student/second year/third year student; ethnicity: African descent/mixed/east Indian descent/unknown; race: mixed/Asian descent/Caucasian descent/East Indian descent/African descent/unknown or prefer not to answer; migration background: no/yes; and past history of sexual intercourse. These variables were typically reported using descriptive statistics, such as frequencies and percentages.

Section B of the questionnaire consisted of 24 items that assessed the respondents' knowledge of HPV and HPV vaccinations. The response options awarded points as follows: yes = 3 points, no = 2 points, and don’t know = 1 point, consistent across all respondents.

In summary, the total knowledge score for each respondent was derived from summing their points across the 24 items. These scores were then categorized to assess the respondents' overall level of HPV and vaccination knowledge.

Section C had ten statements on attitudes toward the HPV vaccine among respondents who were not vaccinated against HPV. It was a 5-point Likert scale ranging from strongly disagree to strongly agree. Strongly agree has a score of 5, agree a score of 4, undecided a score of 3, disagree a score of 2, and strongly disagree a score of 1. In summary, the total attitude score for each respondent was derived from summing their points across the 10 attitude statements. Then, the scores were categorized to assess the general attitudes toward HPV vaccination among respondents.

Section D had 15 statements on intention to obtain the HPV vaccine among respondents who were not vaccinated against HPV. It was a 5-point Likert scale ranging from strongly disagree to strongly agree. Strongly agree has a score of 5, agree a score of 4, undecided a score of 3, disagree a score of 2, and strongly disagree a score of 1. In summary, the total intention score for each respondent was derived from summing their points across the 15 statements, and the scores were categorized to reflect different levels of intention to obtain the HPV vaccine.

A factor analysis of knowledge, attitude, and intention to obtain HPV vaccination with selected social and demographic variables among subjects was done.

Statistical Analysis

Statistical Package for the Social Sciences Statistics version 17 (SPSS Inc., Chicago, IL) was used for data entry, management, and analysis. To minimize data entry errors, check codes were incorporated into the database, and the data were cleaned to ensure consistency of responses.

Data were analyzed using frequencies, means, proportions, standard deviations, and percentages. Factors associated with intention to obtain the HPV vaccination were analyzed using the Spearman rho coefficient. A t-test was performed to statistically analyze overall knowledge and attitudinal scores between genders. Pearson chi-square tests were performed to examine the relationship between genders regarding specific knowledge responses as well as specific attitude responses.

Ethical considerations

All procedures performed in this study for gathering data were in accordance with the ethical standards of the UWI Campus Research Ethics Committee vide ref no: CEC209/05/16.

In addition to the UWI Campus Research Committee approval, permission was also obtained from local authorities in St. Lucia. Specifically, the Sir Arthur Lewis Community College in St. Lucia provided approval to conduct the study, with reference number SALCC/2016/SL4-275/P. This dual-approval process ensured that both institutional and local ethical standards were maintained throughout the research.

## Results

The majority (68 individuals) were female respondents. In the current year of college, there were 56 respondents in their first year, 35 in their second year, and only 9 in their third year of study. Most respondents were of African ethnicity, comprising nearly 59%, followed by those of mixed ethnicity at 32%. A majority, 56%, identified as African race, and 93% reported having no migration background. Nearly 75% of respondents had a history of sexual intercourse (Table 1).

**Table 1:** Classification of respondents according to their demographic characteristics (n = 100)

Parameter	Variable	Frequency	Percentage
Gender	Male	32	32
	Female	68	68
Current year in college	First year	56	56
	Second year	35	35
	Third year	9	9
Ethnicity	African	59	59
	Mixed	32	32
	East Indian	4	4
	Unknown	4	4
	Prefer not to answer	1	1
Race	Mixed	33	33
	Asian	3	3
	Caucasian	2	2
	East Indian	4	4
	African	56	56
	Unknown	2	2
Migration background	No	93	93
	Yes	7	7
Past sexual intercourse	No	25	25
	Yes	75	75

**Figure 2:**
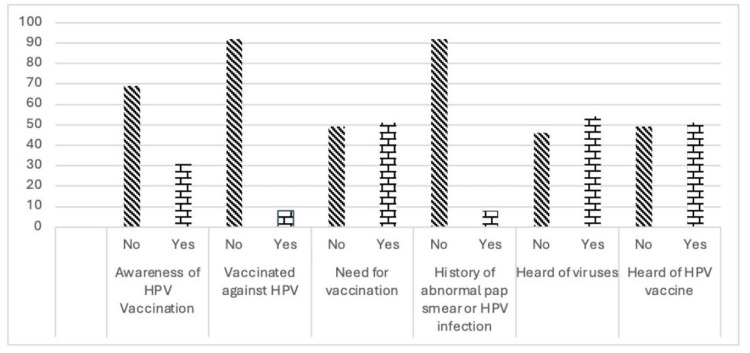
Classification of respondents according to awareness of HPV (n = 100) HPV: human papillomavirus Image credits: This is an original image created by the author Esther S. Daniel

The majority of the respondents, 69%, were unaware of the HPV vaccine, and 92% were not vaccinated against HPV. Half of the participants, 51%, felt the need for vaccination, while 49% did not. Of the participants, 92% had no history of abnormal pap smear or HPV infection, 54% had not heard of the virus, and 46% had heard of it. Roughly 40% of the participants had heard about the HPV vaccine, while 55% had not (Figure 2).

**Figure 3:**
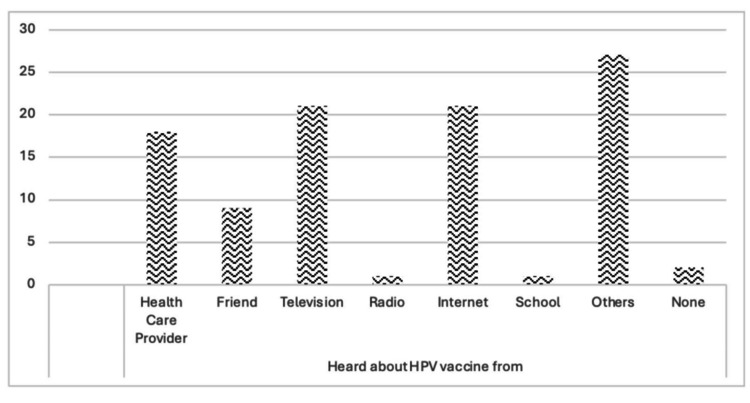
Respondents source of information on HPV (n = 100) HPV: human papillomavirus Image credits: This is an original image created by the author Esther S. Daniel

When respondents were asked from whom they had heard about the HPV vaccine, the majority (27%) said they had heard from others, while 21% said they got information from viewing television and the internet, and 18% said their health care provider informed them. Radio and school had a low percentage of one (Figure 3).

Fifty percent of respondents reported having heard of HPV. Therefore, it can be concluded that there is an urgent need to disseminate information about HPV and HPV vaccination among the vulnerable population.

Knowledge checklist on HPV categories had a score of 2 for "Yes," a score of 1 for "No," and a score of 0 (zero) for "I do not know." Notably, almost half of the respondents felt the need for vaccination and had some awareness of the virus and vaccine. t-tests indicated that knowledge of HPV (1.35 + 0.57), HPV transmission (1.08 + 0.75), HPV causes (0.98 + 0.84), HPV and gender (1.04 + 0.53), preventability of HPV (0.84 + 0.54), and the severity of HPV infection (0.68 + 0.54) were significantly different (p < 0.05) (Table 2).

**Table 2:** Knowledge on HPV vaccination among the respondents (n = 100) ^*^Significant at p < 0.01 HPV: human papillomavirus; SD: standard deviation; STD: sexually transmitted disease

Category	Minimum score	Maximum score	Mean	SD	t-test	p value
Knowledge of HPV	0	2	1.35	0.57	23.45	0.00^*^
HPV an STD	0	2	1.08	0.75	15.62	0.00^*^
HPV causes	0	2	0.98	0.84	21.37	0.00^*^
HPV and gender	0	2	1.04	0.53	19.54	0.00^*^
HPV is preventable	0	2	0.84	0.54	15.97	0.00^*^
HPV infection is serious	0	2	0.68	0.45	15.30	0.00^*^

Respondents were grouped into categories based on score ranges, such as "low," "moderate," and "high" knowledge. The cut-off points for each category were customized (e.g., 0%-25% of the total possible score as "low," 26%-75% as "moderate," and 76%-100% as "high"). t-tests were conducted to explore any significant differences in knowledge scores. The total possible score for each knowledge area was calculated. The score ranges corresponding to "low," "moderate," and "high" knowledge levels were determined. Each mean score was assigned to one of these categories based on its value relative to the total possible score. Each category fell into the "Moderate" knowledge level based on the given mean scores and the categorization criteria: knowledge of HPV: moderate; HPV a sexually transmitted disease (STD): moderate; HPV causes: moderate; HPV and gender: moderate; HPV is preventable: moderate; and HPV infection is serious: moderate. This suggested that the respondents had a moderate level of knowledge across all areas related to HPV. However, it can be inferred that although the respondents had moderate knowledge, it did not motivate them to avail the HPV vaccination.

The study examined the factor analysis of knowledge, attitude, and intention to obtain HPV vaccination with selected social and demographic variables among subjects. As shown in Table 3, the first nine factors have eigenvalues greater than 1. All the factors extracted account for more than 60% of the variance, which is a criterion for factor analysis. Therefore, nine factors were selected.

**Table 3:** Factor analysis of knowledge, attitude, and intention to obtain HPV vaccination with selected social and demographic variables among subjects HPV: human papillomavirus

Component	Initial eigenvalues			Extraction sums of squared loadings		
	Total	% of variance	Cumulative %	Total	% of variance	Cumulative %
1	4.368	16.801	16.801	4.368	16.801	16.801
2	3.332	12.816	29.618	3.332	12.816	29.618
3	2.204	8.476	38.094	2.204	8.476	38.094
4	1.935	7.443	45.537	1.935	7.443	45.537
5	1.693	6.51	52.047	1.693	6.51	52.047
6	1.372	5.276	57.323	1.372	5.276	57.323
7	1.159	4.458	61.781	1.159	4.458	61.781
8	1.101	4.236	66.017	1.101	4.236	66.017
9	1.006	3.868	69.885	1.006	3.868	69.885
10	0.956	3.676	73.561	-	-	-
11	0.857	3.296	76.857	-	-	-
12	0.752	2.892	79.749	-	-	-
13	0.742	2.856	82.604	-	-	-
14	0.716	2.755	85.36	-	-	-
15	0.627	2.413	87.773	-	-	-
16	0.525	2.021	89.794	-	-	-
17	0.495	1.903	91.697	-	-	-
18	0.358	1.378	93.074	-	-	-
19	0.342	1.315	94.389	-	-	-
20	0.293	1.125	95.515	-	-	-
21	0.255	0.98	96.495	-	-	-
22	0.241	0.926	97.421	-	-	-
23	0.225	0.865	98.286	-	-	-
24	0.194	0.745	99.03	-	-	-
25	0.148	0.567	99.598	-	-	-
26	0.105	0.402	100	-	-	-

In the first component matrix, factor 1 was correlated with two variables for which "factor loading value is >0.3," i.e., "I think the risk that HPV can cause diseases like cancer is too little and I think the HPV vaccine is unsafe," factor 2 was correlated with two variables, i.e., "If a teenage girl gets the HPV vaccine, she may be more likely to have sex" and "The HPV vaccine is effective in preventing genital warts," factor 3 was correlated with "I don’t have enough information about the HPV vaccine to decide whether to take it or not," "The risk that HPV can cause diseases like cancer was too little," and "The HPV vaccine might cause short-term problems, like fever or discomfort," factor 4 was correlated with "The HPV vaccine might cause short-term problems like fever or discomfort," factor 5 was correlated with "The HPV vaccine might cause short-term problems like fever or discomfort," "The HPV vaccine was being pushed to make money for drug companies," and "The HPV vaccine is effective in preventing genital warts," factor 6 did not show any correlation as none of the factor loading value is >0.3," factor 7 is correlated with "The HPV vaccine is being pushed to make money for drug companies," factor 8 was correlated with "If a teenage girl gets the HPV vaccine, she may be more likely to have sex," and factor 9 did not show any correlation. So we can conclude that factors 7 and 9 do not explain any variance with the above variables (see Table 4).

**Table 4:** Factor analysis of knowledge, attitude, and intention to obtain HPV vaccination ^a^Eigenvalues above 1 HPV: human papillomavirus; STD: sexually transmitted disease

Component matrix^a^	Component								
	1	2	3	4	5	6	7	8	9
The HPV vaccine might cause short-term problems, like fever or discomfort	-0.057	0.198	0.512	0.337	0.5	0.193	0.238	-0.183	0.089
The HPV vaccine is being pushed to make money for drug companies	-0.279	0.165	-0.098	0.047	0.481	0.296	-0.366	-0.104	0.296
If a teenage girl gets the HPV vaccine, she may be more likely to have sex	-0.081	0.546	0.168	0.026	0.138	0.208	-0.131	-0.33	0.129
I think the HPV vaccine is unsafe	-0.599	-0.006	-0.094	0.272	-0.047	0.274	0.291	-0.232	-0.172
The risk that HPV can cause diseases like cancer is too little	-0.481	-0.017	0.568	0.005	-0.265	0.099	-0.151	0.117	0.046
I do not have enough information about the HPV vaccine to decide whether to take it or not	-0.282	0.105	0.561	0.055	-0.018	-0.013	-0.266	0.102	0.017
The HPV vaccine is effective in preventing genital warts	0.132	0.689	0.181	-0.21	-0.334	0.148	-0.019	-0.03	-0.027
I believe that the vaccine has too many side effects	-0.451	0.191	0.093	0.033	-0.039	-0.179	0.02	0.589	0.124
I’m, in general, against vaccination	-0.357	0.151	0.038	0.398	0.077	0.146	0.658	0.276	0.02
I plan to get vaccinated next year	0.495	-0.416	0.071	-0.046	-0.008	0.151	0.058	0.202	0.081
I understand the severity of STDs	0.364	-0.063	-0.017	-0.368	0.595	-0.04	0.203	0.254	0.198
I understand the severity of cancer	0.578	-0.008	0.17	-0.435	0.064	0.049	0.115	0.095	0.211
I intend to obtain an injection for STD prevention	0.599	-0.135	0.262	-0.14	-0.246	0.422	0.091	-0.197	0.139
I intend to obtain an injection for cancer prevention	0.642	-0.278	0.359	-0.044	-0.154	0.361	0.086	0.077	0.048
I am concerned about the pain at the vaccine injection	-0.452	-0.404	0.355	0.279	-0.046	0.153	-0.302	0.23	0.178
I understand the benefit of the STD prevention injection	0.522	-0.088	-0.161	0.475	0.152	0.157	-0.289	0.286	-0.1
I understand the benefit of the cancer prevention injection	0.543	-0.235	-0.157	0.527	0.064	0.198	-0.163	0.091	-0.103
I have knowledge of HPV	0.37	-0.085	-0.266	0.425	-0.028	-0.204	-0.108	-0.123	-0.06
High-risk HPV causes STDs	0.239	0.451	-0.202	0.086	-0.535	0.118	-0.04	0.064	0.195
Low-risk HPV causes genital cancer	0.064	0.311	-0.378	0.373	-0.323	0.211	0.155	0.095	0.358
Genital warts cause cervical cancer	0.273	0.773	-0.043	-0.142	-0.036	-0.089	-0.065	0.226	-0.104
Cervical cancer is preventable	0.382	0.748	0.181	0.144	0.19	-0.136	-0.045	0.069	-0.051
Genital warts are preventable	0.357	0.605	0.223	0.307	0.171	-0.175	-0.006	-0.051	-0.081
The HPV vaccine prevents STDs	0.064	-0.214	0.491	0.28	-0.257	-0.54	0.104	-0.164	0.27
The HPV vaccine prevents cervical cancer	0.621	-0.212	0.197	0.263	-0.003	-0.398	0.084	-0.144	0.176
After completing this survey, how interested are you in learning more about the HPV vaccine?	-0.269	0.048	-0.433	-0.011	0.069	-0.117	-0.057	-0.103	0.622

Component Matrix

This matrix shows the factor loadings of each variable on the components (factors) extracted during the analysis. Factor loadings represent the correlation between each variable and the extracted component.

Superscript "a"

The "a" superscript typically points to clarifications, conditions, or specific notes about the matrix, such as the extraction method used in the analysis. Component matrix "a" explains that the criteria used for factor extraction are eigenvalues above 1.

In the second component matrix, the first factor correlated with all the variables as the factor loading value is >3. Factor 2 correlated with "I am concerned about pain at vaccine injection" and "I plan to get vaccinated next year," factor 3 correlated with "I Intend to obtain injection for cancer prevention" and "I am concerned about pain at vaccine injection," factor 4 correlated with "I understand the benefit of STD prevention injection," "I understand the benefit of cancer prevention injection," "I’m in general against vaccination," "I understand the severity of STDs," and "I understand the severity of cancer," factor 5 correlated with "I understand the severity of STDs," factor 6 correlated with "I Intend to obtain injection for STD prevention" and "I Intend to obtain injection for cancer prevention," factor 7 correlated with "I am concerned about pain at vaccine injection" "I’m in general against vaccination," factor 8 correlated with "I believe that the vaccine has too many side-effects," and factor 9 did not show any correlation.

In the third component matrix, factor 1 correlated with "I have knowledge of HPV," "Cervical cancer is preventable," "Genital warts are preventable," and "HPV vaccine prevents cervical cancer," factor 2 correlated with "Genital warts are preventable," "Cervical cancer is preventable," "Genital warts cause cervical cancer," "Low-risk HPV causes genital cancer," and "High-risk HPV causes STD," factor 3 correlated with "Low-risk HPV causes genital cancer," "HPV vaccine prevents STDs," and "After completing this survey, how interested are you in learning more about the HPV vaccine?," factor 4 correlated with "Genital warts are preventable," "Low-risk HPV causes genital cancer," and "I have knowledge of HPV," factor 5 correlated with "High-risk HPV causes STD," and "Low-risk HPV causes genital cancer," factor 6 correlated with "HPV vaccine prevents cervical cancer," "HPV vaccine prevents STDs," factors 7 and 8 did not show any correlation, and factor 9 correlated with "Low-risk HPV causes genital cancer" and "After completing this survey, how interested are you in learning more about the HPV vaccine?"

**Figure 4:**
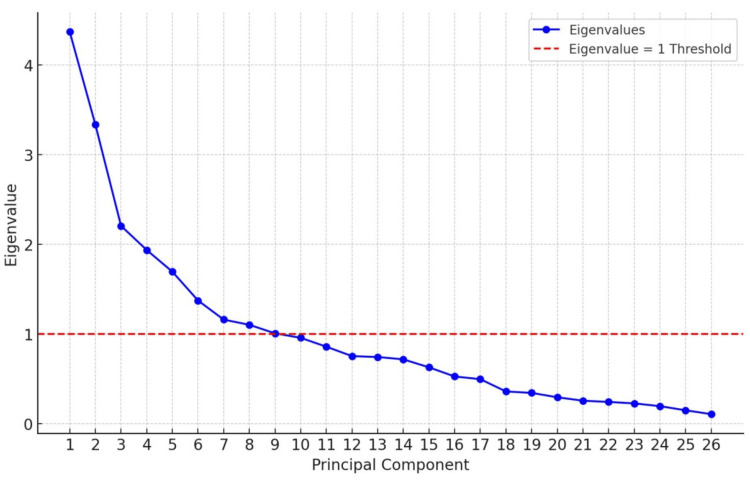
Intention to obtain HPV vaccination with selected social and demographic variables HPV: human papillomavirus Image credits: This is an original image created by the author Esther S. Daniel

A component matrix was conducted for all nine factors, and except for factors 6 and 9, all factors exhibited correlations. The scree plot (Figure 4) indicated that nine factors explain most of the variability, with the slope changing after two variables, suggesting that the initial two factors account for most of the variance.

## Discussion

While the majority of participants in the current study (51%) had heard of the HPV vaccine, a sizeable portion (48%) had not. This discovery accurately represents St. Lucians' understanding of HPV and familiarity with the HPV vaccine. For instance, one study highlighted that most subjects (69%) admitted that they were unaware of HPV immunizations. This result undoubtedly illuminates the study's background [7].

Sixty-nine percent (69%) were unaware that the HPV vaccine was for both sexes. Only 31% knew that the vaccine was for both sexes. This highlights the stereotype in wider society that the HPV vaccine is only for women. This demonstrates the need for a wide-scale educational campaign for citizens. Increased emphasis must be placed on male reproductive health. The current study's findings show that most participants, with no significant difference between genders, understand that HPV affects both sexes. The study revealed that 53% of participants saw a dire need for vaccination against HPV. Twenty-three percent of the participants believed that the HPV vaccine is for sexually active persons. Most respondents (41%) did not know the category of people for which the vaccine was most suited. Only 36% of respondents stated that the vaccine was for the sexually active. This places the targeted population at great risk for contracting HPV, unaware that sexually active and nonsexually active persons may receive the vaccine.

Additionally, most of both genders agreed or strongly agreed that future HPV vaccination campaigns should publicize the fact that the HPV vaccine is for both men and women to create a sense of necessity for men to obtain the vaccine. Past studies have found similar findings, which stated that, in terms of ethics, immunizing boys as well would be just, equal, and would not discriminate against a segment of the population [5].

Most participants (54%) were undecided about HPV vaccine safety, and 37% did not think the vaccine was unsafe. According to earlier research, female students tended to know more about HPV and the vaccine than male students [8]. This is an indicator that participants have a generally positive attitude toward vaccination. This could imply that with mass media coverage and sensitization, citizens can be convinced of the safety and benefits of HPV vaccination. The findings might be related to a study wherein 71% of the 144 students in the study were female students; 37.5% of individuals said they discovered HPV through social media. Eighty-two percent of female and 25% of male students have knowledge about HPV [9].

For the questions "I know at what age to get vaccinated; I know there are three doses of the HPV vaccine and have heard of HPV," the respondents answered "yes"; 71.43% were female and 28.57% were male respondents. This indicates that female respondents were more knowledgeable about the details of the HPV vaccine and its doses. Thus, more educational programs are needed to educate male respondents on these aspects.

Previous studies found that most study participants believed the HPV vaccine to be low risk. However, female students were more likely to share this opinion than male students, who were more inclined to believe that the hazards of HPV infection outweighed those of the vaccine [8]. Out of 100 respondents, 75% of participants (n = 75) self-reported having had a past sexual history, and 25% of respondents (n = 25) did not have a past sexual history. A significant finding is that out of the 75 participants with a past sexual history, seven were vaccinated against HPV. This indicates that 68 participants with a past sexual history are at risk or have been exposed to HPV and are not vaccinated. In addition, 8% of respondents (n = 8) who completed the survey self-reported having an abnormal Pap smear or HPV infection in their lifetime. None of these eight participants had been vaccinated against HPV. Also revealed by this study was the fact that there was no significant correlation between participants' gender, their knowledge of what HPV is, and their opinion on the need for HPV vaccination. Another significant finding is that there was a significant positive correlation between the age of participants and past sexual history. Thus, past sexual history is strongly correlated with the age of participants. This indicates that most of our youngsters in St. Lucia, by the age of 16 years or earlier, are already sexually active. Due to this, the need for all of them to be vaccinated against HPV is dire. For instance, one study highlighted the likelihood that male students would think that HPV vaccination would enhance sexual activity, and unsafe sexual behavior was much higher [8].

Discussion Related to Knowledge and Attitude to Obtain HPV Vaccination

When averaged attitudinal data between male and female participants were compared, the data collected were virtually equivalent and highly favored positive attitudes toward the HPV vaccine.

The statistics acquired were essentially equal when comparing the average sentiments of male and female participants, and they strongly indicated favorable attitudes regarding the HPV vaccine. This was highlighted by a study where participants under the age of 22 were less likely to consent to the vaccination [11]. This study and others highlight the need for further research to determine the acceptability of the HPV vaccine and HPV screening. In addition, peer education, particularly for both sexes in this age group, may be a valuable tool in disseminating vaccine information and increasing acceptability, whether through audiovisual or in-person methods.

Sixty-nine percent of participants were unaware that the HPV vaccine was for both sexes. Only 31% knew that the vaccine was for both sexes. This highlights the stereotype in wider society that the HPV vaccine is only for female participants. This demonstrates the need for a wide-scale educational campaign for citizens. Increased emphasis must be placed on male reproductive health. These findings lead to investigating what can be done to increase the wider society's knowledge of HPV vaccination for men. Although 40% have heard of HPV, 69% were unaware that there is a vaccination against HPV. Also, a highly significant correlation was noted between participants who knew at what age to get vaccinated and those who knew that three doses of the HPV vaccine were required to obtain the full regimen.

Many participants (54.84%) in this survey did not know who the vaccine was for, whereas 32.26% of participants said "yes" that the HPV vaccine was for girls only, and 12.90% said "no" it was not for girls only.

The current study also showed that many participants who heard about HPV and HPV vaccination heard it from the television (34.62%), followed by the internet (26.92%), healthcare providers (23.08%), friends (11.54%), and other forms (3.85%). These findings point out that the television and the internet are the most common places participants have heard about the HPV vaccine; thus, the essential role healthcare providers play in educating individuals about HPV and encouraging them to receive the vaccine is lacking because only 23.08% reported hearing it from a healthcare provider. Furthermore, specific education and workshops for healthcare providers on encouraging youth to be vaccinated may be influential in increasing vaccine uptake and encouraging the Ministry of Health of St. Lucia to see the need for HPV vaccination. Also, a vaccine campaign that focuses on a gender-neutral theme that seeks to normalize getting vaccinated may be a useful approach to increasing uptake.

The current study also assessed attitudes concerning barriers to obtaining the HPV vaccine and shows that 56% of participants are concerned about pain at the injection site after HPV vaccination. Also, 29% are uncertain whether HPV vaccination causes pain, and 15% disagree that it causes pain. This indicates that more must be done to educate the public, as well as in-service workshops or training sessions for healthcare providers on proper administration of injections and ways to abate patients' fears before vaccination. Another significant point is that the cost of this vaccine may be a major barrier to obtaining it, which may be a variable to incorporate in future studies or consider during vaccine campaigning. Most participants (54%) were undecided about HPV vaccine safety, and thirty-seven percent did not think the vaccine was unsafe. The minority (9%) believed that the vaccine was unsafe. This is an indicator that participants have a generally positive attitude toward vaccination. This could imply that with mass media coverage and sensitization, citizens can be convinced of the safety and benefits of HPV vaccination.

A previous study indicated that respondents who got an HPV vaccine on a doctor's recommendation are far more likely than those who got one on a friend's recommendation (RII = 0.745 vs. 0.675) [12].

Discussion Related to Intention to Obtain HPV Vaccination

Although the current study is cross-sectional, the survey tool did have some intervention effects on perspectives on the vaccine. Surprisingly, despite the lack of knowledge of the general population and indecisiveness about vaccine safety and side effects, 82% of participants did intend to get vaccinated against HPV. This could be attributed to St. Lucians' positive attitude toward vaccination and measures for disease prevention. A significant number of participants were again undecided (17%). A mere 1% strongly opposed getting the vaccine. After they had completed their participation in this study, they looked for more information on the HPV vaccine (77%). Twenty-two percent were somewhat interested, whereas 1% were not interested. Some participants expressed to the researcher that this study and educational brochures should also be implemented in secondary schools across the Island due to a pressing need for awareness about HPV and HPV vaccination. The most popular reason participants intended to get the HPV vaccination was cancer prevention. This was rejected by 78% of participants, who agreed to obtain the vaccine for cancer prevention. On the other hand, 20% of the participants remained undecided, and a mere 2% did not agree to obtain the HPV vaccine for cancer prevention. This still shows the dire need for educational programs on HPV vaccination and its benefits.

These findings demonstrate how fast attitudes can vary when completing a questionnaire. Increasing interest in the HPV vaccine may just be this easy. Although interest cannot be linked directly to an increase in the participants' intention to receive the vaccine, this factor is certainly a start in moving forward to enhanced vaccine awareness and uptake.

It might just be this simple to raise awareness of the HPV vaccine. Even though interest cannot be directly linked to a rise in participants' intentions to acquire the vaccination, this element is undoubtedly a start in the direction of greater vaccine knowledge and uptake.

This study’s methodological limitations are as follows: the small sample size of 100 limited generalization of the results as it related to the student population of St. Lucia; the data, collected in one community college, cannot necessarily be generalized to St. Lucia to make a generalized assumption about knowledge, practice and attitude of knowledge, attitude, and intention to obtain HPV vaccination; self-reported data: reliance on self-reported data may have introduced biases, such as recall bias or social desirability bias, particularly on sensitive topics like sexual history and health behaviors; and lack of longitudinal data: since this study was cross-sectional, it captured attitudes and behaviors at a single point in time. This limits the ability to assess changes in awareness or behaviors over time, especially as health information evolves.

## Conclusions

Despite recognizing the associated risks, the findings of this study uncovered a considerable deficiency in public health advocacy. First, there is insufficient effort to raise awareness about HPV, which is widespread in St. Lucia and increases the risk of cancer in both men and women. Second, the HPV vaccine is not accessible in the country. Third, the steep prices of the vaccine ($500 US) and HPV test ($275 EC) serve as obstacles for individuals seeking them. Health professionals in St. Lucia do not promote the HPV vaccine as they should, and information on its availability and accessibility is not widely disseminated. The public remains unaware that the vaccine can be obtained, albeit at a price. Advocating for vaccination of the younger population, aged 11-12, is crucial to preventing cancer from developing later in life.

## Figures and Tables

**Figure 5:**
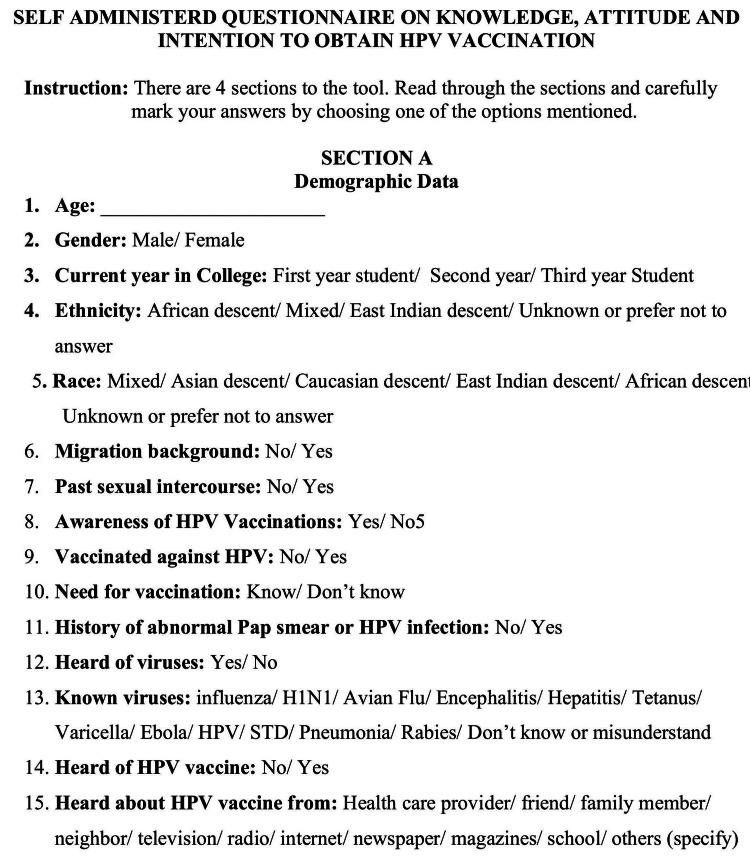
Self-administered questionnaire section A: demographic data

## Appendices

**Table 5:** Section B: knowledge of HPV among the respondents HPV: human papillomavirus; STD: sexually transmitted disease

Sl. no.	Statements	Yes	No	I do not know
1	Heard of HPV	-	-	-
2	HPV is			
2a	A common cold	-	-	-
2b	A skin rash	-	-	-
2c	A sexually transmitted disease	-	-	-
2d	An infection patients get from hospitals	-	-	-
2e	I don’t know what HPV is	-	-	-
3	The most common STD is			
3a	HPV	-	-	-
3b	Chlamydia	-	-	-
3c	Gonorrhea	-	-	-
3d	Herpes	-	-	-
3e	HIV	-	-	-
4	HPV leads to	-	-	-
4a	Cancers of the reproductive system for women (e.g., cervical cancer and vaginal cancer)	-	-	-
4b	Cancers of the reproductive system for men (e.g., penile cancer)	-	-	-
4c	Mouth/throat cancer	-	-	-
4d	AIDS	-	-	-
4e	Genital warts	-	-	-
4f	Breast cancer	-	-	-
4g	None of the above	-	-	-
5	HPV affects both men and women	-	-	-
6	Men can get HPV from homosexual or heterosexual sex	-	-	-
7	You would always know if you had an HPV infection	-	-	-
8	You can get HPV by not keeping yourself clean	-	-	-
9	Antibiotics can cure HPV	-	-	-
10	If you wait to have sex until after marriage, you cannot get HPV	-	-	-
11	HPV is preventable	-	-	-
12	I know at what age to get vaccinated	-	-	-
13	I know there are three doses of the HPV vaccine	-	-	-
14	Both males and females can get the HPV vaccine	-	-	-
15	One must be an adult to get the HPV vaccine	-	-	-
16	When you get the HPV vaccine, you don’t need to use condoms because you are protected from sexually transmitted diseases	-	-	-
17	HPV infection is serious	-	-	-
18	The HPV vaccine is effective in preventing HPV infection	-	-	-
19	The HPV vaccine protects against only genital warts in men	-	-	-
20	Currently, men cannot be tested for HPV	-	-	-
21	The HPV vaccine seems like a vaccine for girls only	-	-	-
22	The HPV vaccine can help prevent cervical cancer	-	-	-
23	The HPV vaccine can prevent genital warts	-	-	-
24	The HPV vaccine is only for people who are sexually active	-	-	-

**Table 6:** Section C: attitudes toward HPV vaccination among respondents HPV: human papillomavirus

Sl. no.	Statements	Strongly agree (5)	Agree (4)	Undecided (3)	Disagree (2)	Strongly disagree (1)
1	The HPV vaccine might cause short-term problems, like fever or discomfort	-	-	-	-	-
2	The HPV vaccine is being pushed to make money for drug companies	-	-	-	-	-
3	If a teenage girl gets the HPV vaccine, she may be more likely to have sex	-	-	-	-	-
4	I think the HPV vaccine is unsafe	-	-	-	-	-
5	The risk that HPV can cause diseases like cancer is too low	-	-	-	-	-
6	I don’t have enough information about the HPV vaccine to decide whether to take it or not	-	-	-	-	-
7	The HPV vaccine is effective in preventing genital warts	-	-	-	-	-
8	I believe that the vaccine has too many side effects	-	-	-	-	-
9	I’m, in general, against vaccination	-	-	-	-	-
10	I plan to get vaccinated next year	-	-	-	-	-

**Table 7:** Section D: intention to obtain HPV vaccination among respondents HPV: human papillomavirus; STD: sexually transmitted disease

Sl. no.	Statements	Strongly agree (5)	Agree (4)	Undecided (3)	Disagree (2)	Strongly disagree (1)
1	I understand the severity of STDs	-	-	-	-	-
2	I understand the severity of cancer	-	-	-	-	-
3	I Intend to obtain injection for STD prevention	-	-	-	-	-
4	I Intend to obtain injection for cancer prevention	-	-	-	-	-
5	I am concerned about pain at vaccine injection	-	-	-	-	-
6	I understand the benefit of STD prevention injection	-	-	-	-	-
7	I understand the benefit of cancer prevention injection	-	-	-	-	-
8	I have knowledge of HPV	-	-	-	-	-
9	High-risk HPV causes STD	-	-	-	-	-
10	Low-risk HPV causes genital cancer	-	-	-	-	-
11	Genital warts cause cervical cancer	-	-	-	-	-
12	Cervical cancer is preventable	-	-	-	-	-
13	Genital warts are preventable	-	-	-	-	-
14	HPV vaccine prevents STDs	-	-	-	-	-
15.	HPV vaccine prevents cervical cancer	-	-	-	-	-

## References

[1] Glasgow L, Lewis R, Charles S (2022). The cancer epidemic in the Caribbean region: further opportunities to reverse the disease trend. Lancet Reg Health Am.

[2] Torres-Roman JS, Ronceros-Cardenas L, Valcarcel B (2022). Cervical cancer mortality among young women in Latin America and the Caribbean: trend analysis from 1997 to 2030. BMC Public Health.

[3] Kasymova S, Harrison SE, Pascal C (2019). Knowledge and awareness of human papillomavirus among college students in South Carolina. Infect Dis (Auckl).

[4] George C, Roberts R, Brennen D, Deveaux L, Read SE (2020). Knowledge and awareness of human papillomavirus (HPV) and HPV vaccines among Caribbean youth: the case of the Bahamas. Hum Vaccin Immunother.

[5] Piñeros M, Laversanne M, Barrios E, Cancela MC, de Vries E, Pardo C, Bray F (2022). An updated profile of the cancer burden, patterns and trends in Latin America and the Caribbean. Lancet Reg Health Am.

[6] Linertová R, Guirado-Fuentes C, Mar-Medina J, Teljeur C (2022). Cost-effectiveness and epidemiological impact of gender-neutral HPV vaccination in Spain. Hum Vaccin Immunother.

[7] Zhang Y, Wang Y, Liu L, Fan Y, Liu Z, Wang Y, Nie S (2016). Awareness and knowledge about human papillomavirus vaccination and its acceptance in China: a meta-analysis of 58 observational studies. BMC Public Health.

[8] Tursun S, Yücel H, Altinel Açoğlu E (2022). Parent’s attitude and knowledge on HPV vaccination: a descriptive study. Turk Hij Den Biyol Derg.

[9] Khan M (2021). Knowledge, attitudes, beliefs and willingness to recommend human papillomavirus (HPV) vaccination among medical students in Mysore, India. Int J Prev Curat Community Med.

[10] Aksoy N, Ozturk N, Ulusoy S, Ömür MF (2022). Knowledge and attitude of students studying at health department towards HPV and HPV vaccination. Vaccine.

[11] El Khoury J, Halabi R, Hleyhel M, El Rahman Kishly W, El Khoury R, Saleh N (2023). HPV vaccination prevalence among Lebanese female university students: a cross-sectional study. J Environ Public Health.

[12] Shetty S, Prabhu S, Shetty V, Shetty AK (2019). Knowledge, attitudes and factors associated with acceptability of human papillomavirus vaccination among undergraduate medical, dental and nursing students in South India. Hum Vaccin Immunother.

[13] Alshammari F, Khan KU (2022). Knowledge, attitudes and perceptions regarding human papillomavirus among university students in Hail, Saudi Arabia. PeerJ.

[14] Becker MH, Maiman LA (1975). Sociobehavioral determinants of compliance with health and medical care recommendations. Med Care.

[15] Rosenstock IM, Strecher VJ, Becker MH (1988). Social learning theory and the Health Belief Model. Health Educ Q.

